# Supplementary material for: Determining structures of RNA conformers using AFM and deep neural networks
Source: Nature. 2024 Dec 18;637(8048):1234–43. doi: 10.1038/s41586-024-07559-x (PMC11779638; doi:10.1038/s41586-024-07559-x)
Supplement: Supplementary file 1 — Supplementary Methods, Tables and a figure. [file 41586_2024_7559_MOESM1_ESM.docx]

**Supplementary Information**

The contents of this file include Supplementary Methods, Supplementary Tables, and a figure.

# Supplementary Methods

**Recapitulation of 3D RNA 3D topological structures from particle AFM images**

The AFM images of individual molecules are digitized into molecular surfaces *Z(x,y)*, where *Z* is the height at the *(x,y)* position. A differentiable cross-correlation function ${CC}^{AFM}(x,y,z)$ (Eq. 1), the corresponding pseudopotential $V^{AFM}\left( x,y,z \right)$(Eq. 2) and the backside potential $B^{stage}\left( x,y,z \right)$ (Eq. 3) are developed and used for dynamic fitting^74^.

${CC}^{AFM}(x,y,z)=\frac{\sum_{i} I_{i}^{exp}I_{i}^{sim}(x,y,z)}{\sqrt{\sum_{i} {{(I}_{i}^{exp})}^{2}}\sqrt{\sum_{i} {(I_{i}^{sim}\left( x,y,z \right))}^{2}}}$ (1)

$V^{AFM}\left( x,y,z \right)={}^{AFM}Nk_{B}T[1-{CC}^{AFM}\left( x,y,z \right)]$ (2)

$B^{stage}\left( x,y,z \right)=\sum_{i}^{N} 4\varepsilon\left[ \left( \frac{d_{i}}{{2z}_{i}} \right)^{12}-\left( \frac{d_{i}}{{2z}_{i}} \right)^{6} \right]$ (3)

where ${CC}^{AFM}\left( x,y,z \right),$ $I_{i}^{exp}$ and $I_{i}^{sim}$ (*x,y,z*) are the cross-correlation, experimental and back-calculated heights, respectively, of the *i^th^* pixel at the (*x,y*) position on the molecular surface; ${}^{AFM}$ is an empirical scaling factor of the AFM force potential $V^{AFM}$; *N* and $k_{B}$ are the total number of beads in the molecule (three beads per residue for RNA: sugar, phosphate, and nucleobase) and the *Boltzmann* constant, respectively; $B^{stage}\left( x,y,z \right)$ is the backside information in the form of Lennard-Jones potential that parameterizes the interaction between the particles on the molecule’s backside and the mica surface; *z_i_* and *d_i_* are the *Z* position and interparticle distance of particle *i*, respectively, and $\varepsilon$ is the interaction-energy factor. The algorithm for simulating the tip dilation effect^75^ is used with the set of empirically determined parameters. $B^{stage}\left( x,y,z \right)$ ensures that the backside of a molecule is in contact with the mica surface, a mere reflection that the molecule must be immobilized to be imaged. Eq. 1 to 3 encapsulates the topological information of both the front and back of a molecule. Dynamic fitting is driven by $V^{AFM}\left( xyz \right)$ and $B^{stage}\left( x,y,z \right)$, and it may achieve the apparent best fit to the molecular surface with a near-perfect correlation score at the expense of both the primary and secondary structures, resulting in a severely distorted RNA 3D structure underneath. To resolve this problem, energy terms, *E^c^*(covalent energy) and *E^nc^* (non-covalent energy) that enforce the RNA hierarchical folding principle are applied together with the AFM pseudopotential (Eq. 1 to 3):

$E^{total}=V^{AFM}\left( x,y,z \right)+{}^{c}\sum\sum E^{c}+\sum{}^{nc(i)}\sum E^{nc}$ (4)

where total energy $E^{total}$is the system energy for the molecule; ${}^{c}\sum\sum E^{c}$ is the covalent energy term that includes bond lengths, angles, and dihedrals:

${}^{c}\left( \sum_{j} E_{j}^{angle}+\sum_{k} E_{k}^{length}+\sum_{l} E_{l}^{dih} \right)$ (5)

whereas the noncovalent energy term $\sum{}^{nc(i)}\sum E^{nc}$ includes stacking, base-pairing, short- and long-range interactions, specifically van der Waals and electrostatic interactions:

$\left( {}^{stacking}\sum_{m} E_{m}^{stacking}+{}^{pairing}\sum_{n} E_{n}^{pairing}+{}^{contact}\sum_{o} E_{o}^{contact} \right)$ (6)

*θ ^AFM^*, *θ ^c^*, *θ ^stacking^, θ ^pairing^* and *θ* ^contact^ are the scaling factors for AFM, covalent, and secondary structural interactions including stacking, base-pairing, and contacts, respectively. We usually set *θ ^contact^* to unity to avoid bias towards the initial structure, whereas *θ ^AFM^*, *θ ^c^*, *θ ^stacking^ and θ ^pairing^* are empirically determined to achieve the optimal balance between enforcing the integrity of primary and secondary structures (the hierarchical principle) and achieving the best fit to the topological restraints at the same time (Eq. 1 to 3). In this study, we found that the optimal empirical values were *θ ^c^* = 5, *θ ^stacking^ = 9 and θ ^pairing^* = 9. Although we expect this set of values to be universally applicable, it is possible that the use of different weighting factors may improve results in specific cases. For such cases, we have provided a script (with instructions in the README) for training HORNET with different scaling factors. The optimal value for *θ ^AFM^* is dependent on the topography of the particle, the noise level of an image, and the closeness of the initial structural model to the “true” structure underneath the molecular surface. We scan through dynamic fitting calculations with *θ ^AFM^* from 2 to 50. In summary, the topological restraint imposed by Eq. 1 to 3 together with the energetics of the primary, secondary and tertiary structures significantly reduces the degrees of freedom that a molecule can sample in a coarse-grained MD trajectory.

Dynamic fitting to low-resolution AFM images of proteins using CafeMol as the platform to obtain relative orientation/position has been demonstrated^74,75^. Implementing the dynamic fitting to high-resolution AFM images for the topological structure determination of RNA, however, involves several practical challenges. First, the CG force field for RNA is far less developed compared to that for proteins, and the relative weights, ${}^{AFM}$, ${}^{c}$, and ${}^{nc}$ of the various energy terms must be empirically determined by following the RNA hierarchical folding principle^76^. Thus, the AFM weighting factor ${}^{AFM}$ is systematically evaluated to obtain an optimal CC, where the primary covalent geometry and secondary structural interactions of the RNA are strictly maintained, and any tertiary interactions present in the initial model are weighted minimally. The direct outcome of this approach is the optimal fit to the experimental AFM topographic surfaces while maintaining the RNA’s primary and secondary structures and avoiding bias toward the initial structure. We developed an algorithm using unsupervised learning (UML) and deep neural networks (DNN) that address this issue and it is able to select and predict the accuracy of the calculated RNA structure by determining a suitable *θ^AFM^* value that would result in an RNA structure closest to the AFM image, without violating covalent and secondary structural restraints.

**Setting up the calculations**

We developed a software pipeline that seamlessly performs all meticulous workflow steps for preparing the input PDB coordinates and the input files. We used Go potential for local interactions and native-contact pairs, Debye-Hückel-type for electrostatic interactions, 15 mM KCl, and a constant temperature of 298.0 K for all structure calculations. For de novo structure determination without a known initial structure, we have adapted the program, RNA2D3D^77,78^, in the form of a singularity container to generate an open input 3D structure from the given secondary structure information. If the coordinates of a conformationally homologous structure, such as a crystal structure, are known, they are used as the initial structure. The dimensions of the processed AFM data are first converted to angstroms. The AFM image is then padded with an area of zero noise around the image to allow enough molecular freedom for translation/rotation during dynamic fitting. Another crucial step in the dynamic fitting is to recenter the initial PDB coordinates in the field of the AFM image in order to produce a meaningful image correlation between the calculated and reference images. The final steps involve setting up header information, including specifying proper pixel sizes, energy parameters, and dynamic fitting parameters. All these steps are seamlessly carried out by execution of the script available at [https://github.com/PNAI-CSB-NCI-NIH/HORNET](https://gcc02.safelinks.protection.outlook.com/?url=https%3A%2F%2Fgithub.com%2FPNAI-CSB-NCI-NIH%2FHORNET&data=05%7C01%7Cwangyunx%40mail.nih.gov%7Cf6e60f6add454d9a318208db2976c3fa%7C14b77578977342d58507251ca2dc2b06%7C0%7C0%7C638149362285184887%7CUnknown%7CTWFpbGZsb3d8eyJWIjoiMC4wLjAwMDAiLCJQIjoiV2luMzIiLCJBTiI6Ik1haWwiLCJXVCI6Mn0%3D%7C3000%7C%7C%7C&sdata=1a2GZWNJDqIGWsPM%2BOq5PSPxOp4Y%2BL4KlH3GtMnIq0w%3D&reserved=0) providing as input the AFM data, model coordinates, and the “ninfo” file (discussed below).

The optimal weighting factors,*θ ^c^* and *θ ^nc^*, which include individual values for bond lengths, bond angles, dihedral angles, hydrogen bonds, base stacking, and long-range interactions, are empirically determined (5, 5, 1, 1, 1, 9, 9), respectively. *θ ^AFM^* varies depending on image quality and deviations from the initial conformation to the optimized conversion toward the AFM topography. We perform dynamic fitting at various *θ^AFM^* values ranging from 2 to 50. The results are analyzed using UML and/or DNN, once the top cohort is ranked by DNN, which is designed to evaluate the energetic and topography information for each model generated during the dynamic fitting.

It is important to properly set up the “ninfo” file, which mainly consists of the interaction parameters for bond lengths, bond angles, dihedral angles, hydrogen bonding across base pairs, base stacking, and long-range tertiary contacts. The scaling factors in this file are empirically customized for RNA folding based on the hierarchical folding principles of RNA, which essentially states that secondary structural elements fold first, leading to the initiation of tertiary contacts. Therefore, the force constants for secondary structure interactions are kept relatively higher to keep the secondary structural elements intact during dynamic fitting. On this front, our pipeline scripts offer the flexibility to change the weights of the scaling factors: *θ ^c^* and *θ^nc^*, which are associated with bond lengths, bond angles, dihedral angles, hydrogen bonding, and base stacking, and *θ* ^contact^ for long-range interactions that are scaled weakly compared to primary and secondary scaling factors to minimize bias toward the initial structures. In addition, one can define the tertiary contacts in a user-friendly text file, which will then be processed for assimilating into the standard “ninfo” file generated by a python script available at [https://github.com/PNAI-CSB-NCI-NIH/HORNET](https://gcc02.safelinks.protection.outlook.com/?url=https%3A%2F%2Fgithub.com%2FPNAI-CSB-NCI-NIH%2FHORNET&data=05%7C01%7Cwangyunx%40mail.nih.gov%7Cf6e60f6add454d9a318208db2976c3fa%7C14b77578977342d58507251ca2dc2b06%7C0%7C0%7C638149362285184887%7CUnknown%7CTWFpbGZsb3d8eyJWIjoiMC4wLjAwMDAiLCJQIjoiV2luMzIiLCJBTiI6Ik1haWwiLCJXVCI6Mn0%3D%7C3000%7C%7C%7C&sdata=1a2GZWNJDqIGWsPM%2BOq5PSPxOp4Y%2BL4KlH3GtMnIq0w%3D&reserved=0). Those user-defined tertiary interactions may be obtained from other biochemical and biophysical measurements.

**Bead to all-atom conversion**

The final coordinates generated from the CG MD calculations are in the form of a three-beads per residue model, which can be rendered into explicit all-atom models. We developed a rendering pipeline that writes out the beads to all atoms and carries out structure refinement using the Xplor-NIH package^79^ . The specific steps for reconstructing the atomistic RNA structures are outlined as follows.

1. Reduction to 1 bead per residue:

[center of mass of 3 beads (xyz)] => [1 bead(hkl)] => apply [all residues]

1. As the majority of RNAs are comprised of A-form duplexes, each duplex region in the RNA is replaced by a matching all-atom duplex built from the standard G2G duplex library. The matching duplex is aligned to the specific location in the RNA based on iterative translation and rotation targeted for best fit. The non-duplex regions are explicitly written out into all-atom coordinates based on references to the bead positions. The steps for writing out the 1-bead-per-residue to the all-atom model can be summarized as:

[duplex regions] => [library based all-atom duplex reconstruction]

[non-duplex regions] => [individual all-atom residue reconstruction]

1. Regularization protocol from Xplor-NIH used to fix covalent geometry.
2. Simulated annealing refinement using Xplor-NIH version 3.1. In addition to the covalent bond, bond angle and improper dihedral terms and the quartic RepelPot term, the following energy terms were employed: base-pair distance and planarity restraints, the TorsionDB statistical torsion angle potential.
3. Torsional angle term, the ORIE base-base positional potential of mean force, and the explicit HBON hydrogen-bonding term. Two non-crystallographic symmetry terms were also employed, one restraining heavy atoms from deviating more than 2 Å RMSD from the input structure, and a second restraining the coordinates to the corresponding 3-bead-per-residue model using the “GlobDiffPot” term with the corresponding energy written as,

$E_{glob}=\frac{1}{2}k_{bead}\sum_{i} \left[ \left| q_{Pi}-r_{Pi} \right|^{2}+\left| q_{Ri}-r_{ri} \right|^{2}+\left| q_{Bi}-r_{Bi} \right|^{2} \right]$ ,

where $k_{bead}$ is an energy scale factor, $q_{i}$ are three positions derived from the current atomic coordinates, $r_{i}$ are the corresponding input bead coordinates, and $i$ is each residue for which bead coordinates are available, summed over all residues. The substitutions P, R, and B represent phosphorous, ring, and base position, respectively, where $q_{Pi}$ is the phosphorous position, $q_{Ri}$ is the average position of the five ribose-ring heavy atoms, and $q_{Bi}$is the position of the N_1_ atom for purine nucleotides and that of the N_3_ atom for pyrimidine nucleotides. For our case studies, we used a value of $k_{bead}$ = 3 kcal/mol throughout the calculations.

**Unsupervised Machine Learning**

**Energy filtering**

This step is performed to remove outliers of unstable conformers generated in the trajectory. The input data is filtered from the whole trajectory (raw data) with *m* total frames (trajectory structures), and the filter function is applied based on the mean value of each *j* energy component:

$\left\langle E_{j} \right\rangle=\left( \sum_{i=1}^{m} E_{j} \right)/m$ (7)

where *j* represents the seven components used: *E^repulsive^, E^local^, E^stacking^, E^pairing^, B^stage^, CC^AFM^ and E^total^*. The frames are filtered by selecting low values of the energy components based on the number of sigma ($n\sigma$) cutoff, where *n* is empirically determined after extensively testing over different simulated data (benchmark 0):

$n\sigma_{E_{j}}=\frac{E_{j}-\left\langle E_{j} \right\rangle}{\sigma_{E_{j}}}$ (8)

All variables are normalized using Eq. 8 and each component now has a mean value centered at zero and described as standard units of σ^80^. The filtering analysis is performed in two steps. First the $n\sigma_{E_{j}}$ threshold is applied to remove outliers from the raw data for the five individual energy components: $E^{repulsive}$, $E^{local}$, $E^{stacking}$, $E^{pairing}$, $B^{stage}$, followed by filtering using $\mathrm{CC}^{AFM}$ and $E^{total}$ cutoffs. The cutoff limits of $n\sigma_{E_{j}}$ are described in **Extended Data Table 8**.

**Principal component analysis (PCA) and clustering**

There are a total of 10 features, which include energetic and topographic information, associated with each frame. We reduce data dimensionality to a small number of components while maintaining the maximum amount of statistical information by applying PCA and clustering. These 10-dimensional vectors are the features describing energetics and topology, the *i*-*th* element of the *m*-*th* structure $\left\{ l_{m}^{i} \right\}:$

$l =\sum_{m}^{i} \boldsymbol{l}_{m}^{i}$ (9)

The vector ***l*** has index m that runs from 1 (first frame) to *m* (last frame) representing all the structure models of the trajectory and all features, which include energy terms *E^total^, E^Go^, E^local^, E^stacking^, E^pairing^, E^repulsive^, E^electrostatic^, B^stage^* and *V^AFM^*, and *CC^AFM^*, after the initial filtering. All $\boldsymbol{l}_{1}^{1}$ $\ldots\boldsymbol{l}_{m}^{10}$vectors could be represented by a data matrix $\boldsymbol{R}(m,10)$. For example, $a_{m,i}$ *m* is the frame of the trajectory and *i* is the energy/topology information:

$R=\left[ \begin{matrix} a_{1,1} & \cdots& a_{1,10} \\ \vdots& \ddots& \vdots\\ a_{m,1} & \cdots& a_{m,10} \end{matrix} \right]=\left[ \begin{matrix} E_{total}^{1} \\ \vdots\\ E_{total}^{m} \end{matrix}\begin{matrix} V_{AFM}^{1} \\ \vdots\\ V_{AFM}^{m} \end{matrix}\begin{matrix} E_{local}^{1} \\ \vdots\\ E_{local}^{m} \end{matrix}\begin{matrix} E_{stacking}^{1} \\ \vdots\\ E_{stacking}^{m} \end{matrix}\begin{matrix} E_{repulsive}^{1} \\ \vdots\\ E_{repulsive}^{m} \end{matrix}\begin{matrix} E_{pairing}^{1} \\ \vdots\\ E_{pairing}^{m} \end{matrix}\begin{matrix} E_{electrostatic}^{1} \\ \vdots\\ E_{electostactic}^{m} \end{matrix}\begin{matrix} B_{stage}^{1} \\ \vdots\\ B_{stage}^{m} \end{matrix}\begin{matrix} E_{Go}^{1} & {CC}^{1} \\ \vdots& \vdots\\ E_{Go}^{m} & {CC}^{m} \end{matrix} \right]$

To derive a linear combination of the 10 features and maximize variance among the components we applied principal component analysis (PCA). In this procedure, we describe our matrix $\boldsymbol{R}$ with another linear combination basis. In other words, we can find a transpose vector $\boldsymbol{\zeta'}$ of constants $\zeta_{1},\zeta_{2},\ldots,\zeta_{10}$ where:

$\boldsymbol{\zeta}_{\boldsymbol{1}}^{\boldsymbol{'}}\mathbf{R}=\zeta_{1}l_{1}+\zeta_{2}\boldsymbol{l}_{1}+\zeta_{3}\boldsymbol{l}_{m}+\ldots+\zeta_{10}\boldsymbol{l}_{1}+\ldots+\zeta_{10}l_{m}$ (10)

However, the Eq. 10 is not a unique solution to describe a linear combination basis of the matrix $\boldsymbol{R}$, other non-correlated solution $\boldsymbol{\zeta}_{\boldsymbol{2}}^{\boldsymbol{'}}\mathbf{R}$ exists, and continues up to the *n*^th^ linear function with $\boldsymbol{\zeta}_{\boldsymbol{n}}^{\boldsymbol{'}}$:

$\boldsymbol{\zeta R=Y}$ (11)

with $\zeta$ the *n*-rows of the linear transformation matrix $\boldsymbol{\zeta}$ that represents the principal components (PC), $\boldsymbol{Y}$ is the matrix ($m \times n$) related product of $\boldsymbol{\zeta R}$*.* The variance presented in this linear combination is quantified using the covariance matrix **S^81^:**

$S_{kj}=\frac{1}{N}\sum_{m=1}^{N} \left( E_{k}^{m}-\left\langle E_{k} \right\rangle\right)(E_{j}^{m}-\left\langle E_{j} \right\rangle)$ (12)

Where *k* and *j* are combinations of the terms of ***l*** (Eq.9). In order to have linear combinations and uncorrelated variables, the covariance matrix needs to be transformed to a diagonal matrix **A**, that is:

$\boldsymbol{P}^{T}\boldsymbol{SP}=\boldsymbol{A}$ (13)

**P** is orthogonal and can be described as a normalized matrix, $\boldsymbol{P}^{T}$ denotes the transpose of $\boldsymbol{P}$. This last equation can be resolved by calculating the eigenvector of the covariance matrix: $\boldsymbol{Sx}=\boldsymbol{x}$, where ***x*** is a vector of **P** with eigenvalues λ, which are the diagonal elements of **A**. In our analysis, this equation is resolved using singular value decomposition (SVD)^82^. Before applying SVD, we standardize all the features using Eq. 8.

To define the number of components describing the trajectory, a primary screening is performed for each dataset to analyze the cumulative variance *versus* the number of components. The general profile for this plot begins with a steep increase in cumulative variance that levels off at a greater number of components. The number of components chosen should be the value just before the cumulative variance plateaus. Using the PCA-space data matrix, the *k*-means algorithm^83^ is applied to separate the data into clusters where each population is defined by the minimization of the inter-cluster entity distances to the geometric center. A similar strategy is used to define the number of clusters for each trajectory, but now the threshold is defined by the correlation plot between the within-cluster sum of squares (WCSS) and the number of clusters. Here, the number of clusters is decided using the first derivative of the WCSS by selecting a value just before the plateau region that still exhibits considerable variations. The outcome is that the representative cluster with the correct structure population should be the one with the structures containing the lowest native energy values. Using that criterion, the *E^local^, E^total^ and E^Go^* energy distributions are analyzed and the cluster with lower mean distribution is selected.

**Cohort model selection**

The cohort of models from the trajectory is selected using the representative cluster. The final selection criteria are applied assuming that the best models must be observed at the highest cross-correlation with the AFM topography ($\mathrm{CC}^{AFM})$ and, at the same time, the energy values need to be populated at the lowest values. A threshold filtering procedure is applied to the representative cluster using the cutoff values described in **Extended Data Table 9**.

**Supervised Deep Neural Networks**

**Data preparation and features**

The dense layers of our DNN are connected by passing the information of each layer forward to the next one, known as a feedforward neural network. The training of a DNN relies on the ability to find the best weights ($W_{j}$) and bias term ($b_{j}$) in all the layers in a way that minimizes the difference between the true value of the feature to be predicted and the output layer ($A_{L}$) of this DNN:

$$A_{1}=g(W_{1}^{T}\cdot X+b_{1})$$

$$A_{2}=g(W_{2}^{T}\cdot A_{1}+b_{2})$$

$$\vdots$$

$$A_{L}=g(W_{L}^{T}\cdot A_{L-1}+b_{L})$$

where $W_{1}$, $W_{2}$, … $W_{L}$ are the weight matrices for the layers 1, 2, … *L*, respectively, containing all the learning weights per feature and per neuron stacked together ($n\times k_{l}$), where *n* is the number of features and $k_{l}$ is the number of neurons in the *l*^th^ layer, while $b_{l}$ is the learning bias term at the *l*^th^ layer, one per neuron ($1\times k_{l}$). The function $g$ is the activation function for this layer, and $A_{l}$ is the result of the activation over the operation on the *l*^th^ layer, which is passed forward to the next layer. $X$ is a matrix of size $n\times m$, where *n* is the number of features and *m* is the number of training examples.

The data were prepared using only the energetic and topographic information. In light of some of the natural correlations among features, whose redundancy could potentially result in overfitting, the features were explored deeply. In the early stages of testing and development, the model was extensively tested through the addition or removal of features, or including combined features. Based on these tests, many original features were removed early on to optimize model performance, resulting in a total of 10 features to train the model. Nine of the features are those used in the UML. These include the energy terms, *E^total^, E^local^, E^go^, E^repulsive^, E^stacking^, E^pairing^, E^eletrostatic^*, the topographic constraint AFM potential $V^{AFM}$, and cross-correlation *CC^AFM^*. However, a new pre-defined feature was added that improved the model convergence and accuracy. This feature is as a combination of two features:

- *CC^AFM^* to the power of *N* x total energy: ${{CC}^{AFM}}^{N}\times$*E^total^*

where *N* = 7 is empirically determined to increase the weight of *CC^AFM^* as a global restraint.

For a generalized model across RNAs with different sizes, the energies *E^total^, E^local^, E^go^, E^repulsive^, E^eletrostatic^* and the potential $V^{AFM}$ were normalized by the number of nucleotides of the RNA. The energies *E^stacking^* and *E^pairing^* were normalized by the number of base-stacking and base-pair interactions. $V^{AFM}$ is further normalized by $\theta^{AFM}$, while *E^stacking^ and E^pairing^* were normalized by $\theta^{stacking}=9$ *and* $\theta^{pairing}=9$, and *E^local^* by *θ ^c^* = 5. After the featurization and normalization, we applied a standard scaling so that the distribution used for training (and testing) would be standardized to allow an optimized convergence of the training. Consequently, all the data used for subsequent RMSD evaluations need to be normalized by the same training normalization parameters to be consistent with the training data and evaluation.

In this study, the output of the DNN was driven to be as close as possible to the RMSD of the training examples by using all the discussed features. The weights and biases for the first layer ($W_{1},b_{1}$) with *k* neurons will be:

$W_{1}=\left[ \begin{matrix} w_{1}^{E^{total}} & w_{2}^{E^{total}} & \cdots& w_{k-1}^{E^{total}} & w_{k}^{E^{total}} \\ w_{1}^{E^{local}} & w_{2}^{E^{local}} & \cdots& w_{k-1}^{E^{local}} & w_{k}^{E^{local}} \\ w_{1}^{E^{go}} & w_{2}^{E^{go}} & \cdots& w_{k-1}^{E^{go}} & w_{k}^{E^{go}} \\ \vdots& \vdots& \vdots& \vdots& \vdots\\ w_{1}^{E^{pairing}} & w_{2}^{E^{pairing}} & \cdots& w_{k-1}^{E^{pairing}} & w_{k}^{E^{pairing}} \\ w_{1}^{V^{AFM}} & w_{2}^{V^{AFM}} & \cdots& w_{k-1}^{V^{AFM}} & w_{k}^{V^{AFM}} \\ w_{1}^{{CC}^{AFM}} & w_{2}^{{CC}^{AFM}} & \cdots& w_{k-1}^{{CC}^{AFM}} & w_{k}^{{CC}^{AFM}} \end{matrix} \right],b_{1}=\left[ \begin{matrix} b_{1} \\ b_{2} \\ \vdots\\ b_{k-1} \\ b_{k} \end{matrix} \right]$ (14)

where each column represents the weights for a single neuron and each row the weight per feature, while *b* is the bias term, one per neuron. The data *X* and the target of the RMSD prediction on the output layer ($\hat{y}$), on the other hand, would be:

$X=\left[ \begin{matrix} x_{1}^{E^{total}} & x_{2}^{E^{total}} & \cdots& x_{m-1}^{E^{total}} & x_{m}^{E^{total}} \\ x_{1}^{E^{local}} & x_{2}^{E^{local}} & \cdots& x_{m-1}^{E^{local}} & x_{m}^{E^{local}} \\ x_{1}^{E^{go}} & x_{2}^{E^{go}} & \cdots& x_{m-1}^{E^{go}} & x_{m}^{E^{go}} \\ \vdots& \vdots& \vdots& \vdots& \vdots\\ x_{1}^{E^{pairing}} & x_{2}^{E^{pairing}} & \cdots& x_{m-1}^{E^{pairing}} & x_{m}^{E^{pairing}} \\ x_{1}^{V^{AFM}} & x_{2}^{V^{AFM}} & \cdots& x_{m-1}^{V^{AFM}} & x_{m}^{V^{AFM}} \\ x_{1}^{{CC}^{AFM}} & x_{2}^{{CC}^{AFM}} & \cdots& x_{m-1}^{{CC}^{AFM}} & x_{m}^{{CC}^{AFM}} \end{matrix} \right], \hat{y}\to y=\left[ \begin{matrix} x_{1}^{RMSD} \\ x_{2}^{RMSD} \\ \vdots\\ x_{m-1}^{RMSD} \\ x_{m}^{RMSD} \end{matrix} \right]$ (15)

where the columns in *X* represent each training example (up to *m* examples) and the rows of each feature. The output layer should predict values for the RMSD as closely as possible to the real ones for all the data examples.

**Loss function**

The process of learning in an artificial neural network (ANN) depends on the loss function. The mean squared error (MSE, also known as L2 loss or quadratic loss) and the Huber loss, had both great performances in our training:

$L_{MSE}=\sum_{i=1}^{m} {(\hat{y}_{i}- y_{i})}^{2}$

$L_{Huber}=\sum_{i=1}^{m} \left[ \begin{matrix} \frac{1}{2}{(\hat{y}_{i}- y_{i})}^{2} \\ \delta\cdot(\left| \hat{y}_{i}- y_{i} \right|-\frac{1}{2}\delta) \end{matrix} \right.\begin{matrix} for \left| \hat{y}_{i}- y_{i} \right|<\delta\\ for \left| \hat{y}_{i}- y_{i} \right|\geq\delta\end{matrix}$ (16)

where $\hat{y}$ is the prediction for a single training sample and $y$ its true value. $\delta$ in the Huber loss sets the region where the loss will assume a squared difference or absolute difference, so it does not overweight the outliers (such as in the MSE loss), nor simplify the loss by the averages. In the training of our DNN model, we used the Adam ^84,85^ optimizer to minimize the loss function.

**Estimation of auto-correlated value of AFM images**

The ACV profiles show some discontinuities, or *kinks*, that are signatures of topographical features (**Fig. 2d**). To be able to distinguish these details, the first derivative of the ACV profile was calculated and shown in **Fig. 2e**.

$ACV=\frac{\sum_{i} {(R}_{i}-\overline{R})\times(R_{i}^{'}-\overline{R^{'}})}{\left\{ \sum_{i} {{(R}_{i}-\overline{R})}^{2}\times\sum_{i} {(R_{i}^{'}-\overline{R^{'}})}^{2} \right\}^{1/2}}$ (17)

where *i* is the (x,y) pixel position with a Z-height (*R*) of the original reference image and a Z-height (R') after applying a low-pass filtered Fourier ring; $\overline{R}$ and $\overline{R^{'}}$ represent the mean values of the Z-heights of reference and low-pass filtered images, respectively.

# Supplementary Tables

**Supplementary Table 1 | Summary of HORNET benchmarking and experimental test cases.** A total of fifteen cases were used to train, validate, and test HORNET. The benchmarks (B0-B5) were designed to test HORNET using various initial structural models and the simulated AFM based on ground-truth structures with known structural information. Two experimental cases were performed with unknown GT structures: the full-length RNase P RNA and the HIV-1 RRE RNA, where we determined three and five distinct conformer structures, respectively.

| Data | RNA | Size | Starting model | AFM data | **RMSD_initial-GT_** |
| --- | --- | --- | --- | --- | --- |
| BM0 | Catalytic domain of RNase P RNA | 268 | Trajectory model with the best ARES score | simulated from pdb id 3DHS | 21.4 |
| BM1 | Catalytic domain of RNase P RNA | 268 | FARFAR prediction S142 | simulated from pdb id 3DHS | 13.5 |
| BM2 | Catalytic domain of RNase P RNA | 268 | FARFAR prediction S1076 | simulated from pdb id 3DHS | 22.3 |
| BM3 | Cobalamin-sensing riboswitch (rCbl) | 210 | derived from experimental AFM data | simulated from pdb id 4GMA | 10.8 |
| BM4 | Group-II intron | 387 | derived from RS3D simulated-SAXS data | simulated from pdb id 4E8K | 16.1 |
| BM5 | RNase P RNA | 298 | derived from RS3D simulated-SAXS data | simulated from pdb id 2A64 | 14.0 |
| S257 | Catalytic domain of RNase P RNA | 268 | FARFAR prediction model with best ARES score | simulated from pdb id 3DHS | 30.0 |
| Case 1:  P1, P2 and P3 | Full-length RNase P RNA | 417 | crystal structure full-length RNase P (2A64) | experimental  image | ---- |
| Case 2:  M0, M1, M2, M3 and M4 | HIV-1 RRE | 233 | derived from experimental SAXS | experimental  image | ---- |

**Notes:** BM0, BM1, BM2, and S257 are the catalytic domain of the RNase P RNA^86^ using different starting models. BM0 is a trajectory model with the best ARES^87^ score (**Extended Data Fig. 6a**, **b**); BM1, BM2, and S257 were selected from an ensemble of 10,000 models generated using FARFAR2^88^, based on ARES score, FARFAR score, or a combination of both. BM3 uses a trajectory structural model from an MD calculation of cobalamin riboswitch^89^. BM4 and BM5 are the topological low-resolution structures of group II intron^90^ and RNase P RNA^91^, respectively, and were derived using RS3D small angle X-ray scattering (SAXS) data^77,92^. These benchmark RNAs represent the majority of classes of naked RNAs larger than 210 residues in the PDB.

**Supplementary Table 2 | Top Models from Benchmark 0.** Top 10 models with the lowest total energy (*E^Total^*) scores, selected from the final cohorts of the Benchmark 0 trajectories with different applied Gaussian noise levels (10–50%) (see Extended Data Fig. 4).

| **Noise level (%)** | **RMSD ( Å )** | ***E^Total^ (kcal/mol)*** | ***θ^AFM^*** | ***CC^AFM^*** |
| --- | --- | --- | --- | --- |
| **10** | 6.845 | -6590.95 | 12 | 0.994 |
|  | 4.175 | -6584.259 | 14 | 0.993 |
|  | 3.851 | -6582.08 | 10 | 0.989 |
|  | 4.946 | -6581.46 | 10 | 0.989 |
|  | 4.208 | -6579.19 | 14 | 0.990 |
|  | 4.22 | -6578.44 | 12 | 0.991 |
|  | 5.172 | -6577.44 | 12 | 0.989 |
|  | 4.558 | -6576.849 | 12 | 0.989 |
|  | 5.946 | -6575.95 | 12 | 0.990 |
|  | 4.179 | -6575.89 | 10 | 0.993 |
| **15** | 4.815 | -6584.679 | 15 | 0.988 |
|  | 4.772 | -6579.11 | 15 | 0.987 |
|  | 4.944 | -6577.17 | 9 | 0.987 |
|  | 3.603 | -6576.71 | 13 | 0.991 |
|  | 3.583 | -6575.46 | 15 | 0.989 |
|  | 4.225 | -6574.59 | 14 | 0.988 |
|  | 4.426 | -6573.31 | 15 | 0.988 |
|  | 4.075 | -6573.0 | 13 | 0.990 |
|  | 4.72 | -6570.820 | 14 | 0.988 |
|  | 4.48 | -6570.71 | 14 | 0.990 |
| **20** | 5.462 | -6578.83 | 6 | 0.982 |
|  | 3.847 | -6578.59 | 12 | 0.986 |
|  | 4.863 | -6578.43 | 7 | 0.982 |
|  | 5.199 | -6577.97 | 12 | 0.982 |
|  | 3.772 | -6577.42 | 12 | 0.982 |
|  | 4.441 | -6574.980 | 12 | 0.982 |
|  | 4.203 | -6573.91 | 12 | 0.984 |
|  | 4.164 | -6573.75 | 12 | 0.983 |
|  | 4.707 | -6573.019 | 6 | 0.983 |
|  | 5.404 | -6572.22 | 12 | 0.981 |
| **30** | 4.565 | -6592.24 | 15 | 0.966 |
|  | 4.362 | -6578.84 | 15 | 0.969 |
|  | 3.278 | -6577.93 | 15 | 0.969 |
|  | 4.277 | -6577.06 | 13 | 0.969 |
|  | 5.052 | -6574.1 | 15 | 0.967 |
|  | 4.929 | -6573.61 | 13 | 0.966 |
|  | 4.384 | -6572.62 | 15 | 0.970 |
|  | 4.657 | -6571.423 | 15 | 0.967 |
|  | 4.239 | -6570.70 | 13 | 0.968 |
|  | 4.641 | -6570.20 | 15 | 0.966 |
| **40** | 6.644 | -6596.84 | 12 | 0.946 |
|  | 4.241 | -6581.09 | 9 | 0.945 |
|  | 4.04 | -6580.07 | 9 | 0.948 |
|  | 4.366 | -6579.37 | 12 | 0.948 |
|  | 5.097 | -6577.94 | 12 | 0.948 |
|  | 4.825 | -6576.86 | 8 | 0.945 |
|  | 4.383 | -6576.62 | 9 | 0.944 |
|  | 4.346 | -6575.33 | 8 | 0.944 |
|  | 4.417 | -6573.34 | 12 | 0.950 |
|  | 4.482 | -6570.54 | 9 | 0.947 |
| **50** | 5.615 | -6575.15 | 8 | 0.922 |
|  | 4.305 | -6574.33 | 8 | 0.924 |
|  | 4.117 | -6570.94 | 10 | 0.920 |
|  | 5.602 | -6570.5 | 11 | 0.924 |
|  | 6.375 | -6570.07 | 10 | 0.919 |
|  | 5.666 | -6567.21 | 11 | 0.926 |
|  | 6.206 | -6566.74 | 10 | 0.923 |
|  | 6.325 | -6566.47 | 10 | 0.923 |
|  | 4.446 | -6564.43 | 7 | 0.922 |
|  | 5.15 | -6563.86 | 10 | 0.921 |

**Supplementary Table 3 | Deep neural network dataset variation, filtering, hyperparameter options, and intervals.**

| **Dataset Composition** | **Possible options** |
| --- | --- |
| Data | BM 0 |
|  | BM 0 + 5% BM 1 |
|  | BM 0 + 5% BM 2 |
|  | BM 0 + 5% BM 1 + 5% BM 2 |
| Max kappa | 15, 25, 50* or not cut |
| BM 0 noises | 5, 10, 5-15*, 5-25 or 5-50% |
| **Hyperparameter** | **Possible values** |
| Number of layers | 1 – 10 |
| Number of neurons | 8 – 256 |
| Neuron activations | relu, leakly-relu, elu*, gelu or selu |
| Regularization | L2, Dropout*, Dropout + L2 or Alpha Dropout (for selu activation) |
| L2 factor | 0.1, 0.03, 0.01, 0.003 or 0.001 |
| Dropout rate | 10, 20* or 50% |
| Optimizer | Adam* or SGD |
| Learning Rate | 0.01, 0.003, 0.001*, 0.0003 or 0.0001 |
| Weight initialization | Glorot Uniform, He Normal* or Lecun Normal |
| Mini-batch size | 16, 32, 64, 128* or 256 |
| Number of epochs | 300 |

**Note**: * indicates the best option found.

**Supplementary Table 4 | Estimated RMSD for the top 10 models selected using HORNET from each of the BM0 UML–cohorts.**

| **AFM Noise**  **Level** | **True RMSD** | ***Predicted*** | **AFM Noise**  **Level** | **True RMSD** | ***Predicted*** | **AFM Noise**  **Level** | **True RMSD** | ***Predicted*** |
| --- | --- | --- | --- | --- | --- | --- | --- | --- |
| 5% | 3.51 | 4.93 | 10% | 4.45 | 4.89 | 15% | 3.99 | 5.01 |
|  | 5.29 | 4.94 |  | 5.44 | 4.94 |  | 5.22 | 5.02 |
|  | 6.07 | 4.94 |  | 5.67 | 4.98 |  | 4.297 | 5.02 |
|  | 3.80 | 4.94 |  | 5.65 | 4.98 |  | 3.84 | 5.02 |
|  | 4.07 | 4.94 |  | 5.47 | 5.00 |  | 3.81 | 5.02 |
|  | 5.98 | 4.95 |  | 4.62 | 5.00 |  | 3.82 | 5.03 |
|  | 5.40 | 4.98 |  | 4.35 | 5.01 |  | 4.079 | 5.03 |
|  | 4.12 | 4.97 |  | 3.57 | 5.01 |  | 3.99 | 5.04 |
|  | 5.6 | 4.98 |  | 3.65 | 5.01 |  | 4.10 | 5.04 |
|  | 5.8 | 4.99 |  | 5.58 | 5.02 |  | 4.61 | 5.05 |

| **AFM Noise**  **Level** | **True RMSD** | ***Predicted*** | **AFM Noise**  **Level** | **True RMSD** | ***Predicted*** |
| --- | --- | --- | --- | --- | --- |
| 20% | 5.12 | 5.05 | 30% | 4.24 | 5.31 |
|  | 3.67 | 5.05 |  | 6.43 | 5.31 |
|  | 3.47 | 5.07 |  | 4.30 | 5.32 |
|  | 4.02 | 5.08 |  | 5.08 | 5.32 |
|  | 3.84 | 5.09 |  | 5.32 | 5.38 |
|  | 4.43 | 5.10 |  | 4.05 | 5.33 |
|  | 5.54 | 5.11 |  | 4.97 | 5.33 |
|  | 3.94 | 5.12 |  | 4.99 | 5.33 |
|  | 4.43 | 5.12 |  | 4.30 | 5.34 |
|  | 5.39 | 5.14 |  | 5.70 | 5.34 |

**Supplementary Table 5 | Top 10 HORNET-derived models for full-length RNase P RNA particles, P1, P2 and P3.** Respectively, values for the radius of gyration (Rg), the energy terms: total (E^Total^), local (E^Local^), Go (E^Go^), repulsive (E^repul^), stacking( E^stacking^), pairing (E^pairing^), and electrostatic (E^elect^), the AFM cross-correlation value between experimental AFM topography and calculated model (CC^AFM^), AFM potential (V^AFM^), AFM force factor (θ^AFM^), and estimated accuracy in terms of RMSD (pred) in Å. All energies are in units of kcal/mol.

| **Particle** | **Rg** | ***E^Total^*** | ***E^Local^*** | ***E^Go^*** | ***E^repul^*** | ***E^stacking^*** | ***E^pairing^*** | ***E^elect^*** | ***CC^AFM^*** | **V^AFM^** | **stage** | ***θ^AFM^*** | **pred** |
| --- | --- | --- | --- | --- | --- | --- | --- | --- | --- | --- | --- | --- | --- |
| P1 | 40.8 | -6604.83 | 917.37 | -802.74 | 2.65 | -4116.24 | -3201.37 | 423.25 | 0.98 | 187.73 | -16.46 | 14 | 5.6 |
|  | 40.8 | -6599.19 | 936.15 | -789.05 | 2.71 | -4128.88 | -3211.06 | 416.36 | 0.98 | 190.64 | -17.06 | 14 | 5.7 |
|  | 41.6 | -6585.88 | 946.85 | -806.8 | 1.87 | -4128.72 | -3204.72 | 414.83 | 0.98 | 205.6 | -15.78 | 14 | 5.7 |
|  | 40.8 | -6582.69 | 961.16 | -794.42 | 2.23 | -4135.77 | -3213.57 | 416.38 | 0.98 | 198.06 | -17.75 | 14 | 5.7 |
|  | 41.1 | -6577.27 | 948.44 | -788.09 | 1.97 | -4129.77 | -3219.5 | 421.94 | 0.98 | 199.77 | -13.01 | 14 | 5.7 |
|  | 40.1 | -6569.6 | 940.3 | -780.57 | 3.05 | -4128.29 | -3213.31 | 416.69 | 0.98 | 205.31 | -13.76 | 14 | 5.7 |
|  | 41.1 | -6625.17 | 919.29 | -808.64 | 2.1 | -4137.25 | -3203.67 | 412.22 | 0.98 | 203.59 | -13.79 | 14 | 5.7 |
|  | 40.8 | -6628.65 | 881.59 | -769.65 | 2.99 | -4124.15 | -3207.14 | 415.82 | 0.98 | 184.55 | -13.64 | 14 | 5.7 |
|  | 41.6 | -6572.29 | 974.5 | -815.71 | 1.3 | -4132.15 | -3212.46 | 412.56 | 0.98 | 214.86 | -16.18 | 14 | 5.7 |
|  | 40.9 | -6580.52 | 928.91 | -776.25 | 2.86 | -4128.89 | -3216.96 | 417.23 | 0.98 | 206.44 | -14.84 | 14 | 5.8 |
| P2 | 49.1 | -9660.63 | 818.99 | -839.05 | 2.81 | -4869.61 | -5463.29 | 455.27 | 0.99 | 243.07 | -9.81 | 24 | 4.4 |
|  | 48.3 | -9595.19 | 838.6 | -866.67 | 2.65 | -4863.44 | -5460.94 | 457.43 | 0.98 | 303.59 | -7.4 | 23 | 4.4 |
|  | 48.3 | -9580.44 | 852.79 | -866.63 | 2.36 | -4858.09 | -5461.75 | 457.68 | 0.98 | 299.15 | -6.92 | 23 | 4.4 |
|  | 48.4 | -9599.43 | 834.6 | -865.72 | 4.05 | -4858.0 | -5466.5 | 457.31 | 0.98 | 299.96 | -6.11 | 23 | 4.5 |
|  | 48.8 | -9613.26 | 832.48 | -834.27 | 2.78 | -4848.85 | -5467.74 | 453.01 | 0.99 | 253.92 | -5.58 | 24 | 4.5 |
|  | 48.8 | -9565.62 | 852.32 | -842.8 | 5.27 | -4857.46 | -5447.19 | 464.11 | 0.99 | 264.71 | -5.57 | 24 | 4.5 |
|  | 48.4 | -9581.44 | 830.72 | -843.33 | 3.61 | -4857.8 | -5468.03 | 456.81 | 0.98 | 301.48 | -5.88 | 23 | 4.5 |
|  | 48.8 | -9568.25 | 847.85 | -838.45 | 4.94 | -4876.16 | -5457.87 | 463.42 | 0.98 | 289.74 | -2.71 | 24 | 4.5 |
|  | 49.1 | -9614.55 | 855.41 | -853.13 | 3.59 | -4856.91 | -5465.01 | 455.33 | 0.99 | 254.86 | -9.68 | 24 | 4.5 |
|  | 49.1 | -9583.19 | 851.66 | -831.68 | 3.25 | -4864.33 | -5468.04 | 461.8 | 0.98 | 269.61 | -6.45 | 24 | 4.5 |
| P3 | 45.7 | -9646.01 | 840.73 | -885.97 | 3.02 | -4870.25 | -5462.91 | 451.66 | 0.98 | 276.73 | 0.0 | 15 | 4.7 |
|  | 45.7 | -9631.0 | 835.74 | -892.34 | 1.58 | -4859.65 | -5469.42 | 452.57 | 0.93 | 299.55 | 0.0 | 15 | 4.7 |
|  | 45.7 | -9644.31 | 829.6 | -882.71 | 3.29 | -4872.97 | -5459.24 | 450.93 | 0.97 | 285.82 | 0.0 | 15 | 4.7 |
|  | 45.7 | -9157.99 | 869.07 | -858.81 | 2.81 | -4864.22 | -5453.21 | 450.76 | 0.98 | 694.64 | 0.0 | 49 | 4.8 |
|  | 45.8 | -9137.16 | 869.85 | -840.52 | 2.83 | -4866.05 | -5456.34 | 450.32 | 0.98 | 701.77 | 0.0 | 49 | 4.8 |
|  | 46.7 | -9132.9 | 879.88 | -852.03 | 2.13 | -4854.5 | -5460.52 | 447.25 | 0.98 | 703.93 | 0.0 | 49 | 4.8 |
|  | 45.9 | -9110.73 | 862.98 | -839.6 | 2.49 | -4874.27 | -5458.91 | 450.52 | 0.98 | 745.08 | 0.0 | 49 | 4.8 |
|  | 45.8 | -9146.07 | 883.73 | -840.65 | 2.4 | -4871.48 | -5472.66 | 452.17 | 0.98 | 699.45 | 0.0 | 49 | 4.8 |
|  | 45.8 | -9083.82 | 872.33 | -853.09 | 3.58 | -4874.93 | -5453.82 | 451.42 | 0.98 | 769.72 | 0.0 | 49 | 4.8 |
|  | 45.8 | -9593.03 | 840.31 | -874.08 | 1.89 | -4859.49 | -5461.77 | 452.17 | 0.97 | 306.96 | 0.0 | 15 | 4.8 |

**Supplementary Table 6 | Top 10 HORNET-derived models selected from the cohort of UML and evaluated by DNN for full-length RNase P RNA particles, P1, P2 and P3.** Respectively, values for the energy terms (in kcal/mol): total (E^Total^), local (E^Local^), Go (E^Go^), repulsive (E^repul^), stacking( E^stacking^), pairing (E^pairing^), and electrostatic (E^elect^), the AFM cross-correlation value between experimental AFM topography and calculated model (CC^AFM^), AFM potential (V^AFM^), AFM force factor (θ^AFM^), and, finally, estimated model accuracy in terms of RMSD (pred), in Å.

| **Particle** | | ***E^Total^*** | ***E^Local^*** | ***E^Go^*** | ***E^repul^*** | ***E^stacking^*** | ***E^pairing^*** | ***E^elect^*** | ***CC^AFM^*** | **V^AFM^** | **stage** | ***θ^AFM^*** | **pred** |
| --- | --- | --- | --- | --- | --- | --- | --- | --- | --- | --- | --- | --- | --- |
| P1 | -6659.61 | | 887.29 | -787.74 | 3.54 | -4140.5 | -3207.66 | 410.04 | 0.98 | 199.73 | -25.29 | 14 | 5.8 |
|  | -6651.64 | | 902.54 | -793.21 | 3.51 | -4142.06 | -3208.7 | 416.63 | 0.98 | 183.98 | -15.32 | 14 | 5.8 |
|  | -6653.47 | | 908.18 | -795.04 | 3.38 | -4132.93 | -3216.59 | 412.33 | 0.98 | 180.53 | -14.31 | 14 | 5.8 |
|  | -6649.23 | | 901.84 | -794.22 | 3.81 | -4141.64 | -3208.87 | 407.68 | 0.98 | 204.87 | -23.69 | 14 | 5.8 |
|  | -6645.38 | | 921.97 | -796.12 | 3.41 | -4141.51 | -3217.44 | 407.74 | 0.98 | 207.62 | -32.04 | 14 | 5.8 |
|  | -6661.07 | | 924.99 | -809.65 | 1.66 | -4150.1 | -3214.15 | 412.13 | 0.98 | 184.23 | -11.16 | 14 | 5.9 |
|  | -6642.3 | | 891.19 | -787.19 | 2.45 | -4136.72 | -3205.11 | 417.22 | 0.98 | 186.4 | -11.51 | 14 | 5.9 |
|  | -6672.08 | | 908.6 | -806.37 | 2.86 | -4139.21 | -3210.55 | 417.97 | 0.98 | 165.22 | -11.58 | 11 | 5.9 |
|  | -6643.87 | | 911.24 | -791.59 | 2.64 | -4135.96 | -3215.72 | 418.27 | 0.98 | 178.15 | -11.88 | 14 | 5.9 |
|  | -6640.0 | | 901.39 | -789.34 | 5.92 | -4133.27 | -3210.17 | 410.79 | 0.98 | 188.3 | -14.61 | 14 | 6.0 |
| P2 | -9744.31 | | 819.73 | -844.03 | 3.59 | -4852.8 | -5468.51 | 451.5 | 0.98 | 157.21 | -11.98 | 12 | 4.6 |
|  | -9741.05 | | 825.74 | -844.93 | 2.92 | -4861.54 | -5460.19 | 447.12 | 0.98 | 158.1 | -9.25 | 12 | 4.7 |
|  | -9798.4 | | 800.0 | -860.37 | 2.93 | -4875.19 | -5472.76 | 446.06 | 0.98 | 169.56 | -9.61 | 10 | 4.7 |
|  | -9741.4 | | 830.51 | -873.88 | 2.01 | -4880.01 | -5462.33 | 445.35 | 0.98 | 206.97 | -11.01 | 15 | 4.7 |
|  | -9757.01 | | 825.43 | -851.19 | 2.78 | -4872.95 | -5470.81 | 447.65 | 0.98 | 173.08 | -11.98 | 10 | 4.7 |
|  | -9738.28 | | 818.26 | -855.04 | 1.75 | -4855.52 | -5458.95 | 445.8 | 0.98 | 174.18 | -9.73 | 12 | 4.7 |
|  | -9738.12 | | 824.34 | -854.36 | 2.88 | -4869.83 | -5465.06 | 446.45 | 0.98 | 188.22 | -11.74 | 12 | 4.7 |
|  | -9743.96 | | 839.65 | -854.56 | 2.33 | -4881.07 | -5466.98 | 445.21 | 0.98 | 178.35 | -7.86 | 12 | 4.7 |
|  | -9773.79 | | 820.63 | -862.18 | 3.1 | -4882.2 | -5469.61 | 447.26 | 0.98 | 176.81 | -8.58 | 10 | 4.7 |
|  | -9740.64 | | 835.96 | -847.86 | 2.76 | -4865.34 | -5472.79 | 447.41 | 0.98 | 168.6 | -10.36 | 10 | 4.7 |
| P3 | -9710.9 | | 814.06 | -870.58 | 2.31 | -4875.42 | -5466.14 | 447.13 | 0.98 | 236.76 | 0.0 | 14 | 4.8 |
|  | -9715.56 | | 823.28 | -880.53 | 2.57 | -4880.06 | -5472.17 | 445.04 | 0.98 | 245.34 | 0.0 | 14 | 4.9 |
|  | -9717.65 | | 794.77 | -875.14 | 2.79 | -4875.21 | -5463.25 | 444.77 | 0.97 | 252.64 | 0.0 | 14 | 5.0 |
|  | -9782.39 | | 850.0 | -881.4 | 2.69 | -4875.07 | -5468.0 | 442.5 | 0.97 | 145.91 | 0.0 | 8 | 5.0 |
|  | -9702.2 | | 825.03 | -871.32 | 3.08 | -4879.89 | -5463.83 | 439.91 | 0.97 | 243.84 | 0.0 | 12 | 5.0 |
|  | -9789.33 | | 820.5 | -890.02 | 2.36 | -4861.36 | -5458.24 | 443.22 | 0.98 | 153.24 | 0.0 | 8 | 5.0 |
|  | -9777.07 | | 868.14 | -896.49 | 2.76 | -4867.45 | -5464.51 | 441.46 | 0.98 | 138.04 | 0.0 | 8 | 5.0 |
|  | -9703.17 | | 826.41 | -881.08 | 2.66 | -4862.83 | -5469.57 | 444.79 | 0.98 | 235.46 | 0.0 | 14 | 5.0 |
|  | -9706.74 | | 820.8 | -883.63 | 2.81 | -4868.88 | -5450.65 | 442.88 | 0.98 | 228.96 | 0.0 | 14 | 5.0 |
|  | -9756.69 | | 861.67 | -874.73 | 2.12 | -4873.94 | -5459.67 | 440.92 | 0.98 | 145.96 | 0.0 | 8 | 5.0 |

**Supplementary Table 7 | Top 10 HORNET-derived models selected from the cohort of UML and evaluated by DNN for particles C1, C2, C3, C4, and C5, of RRE RNA.** Respectively, values for the energy terms (in kcal/mol): total (*E^Total^*), local (*E^Local^*), Go (*E^Go^)*, repulsive (*E^repul^)*, stacking (*E^stacking^*), pairing (*E^pairing^*), and electrostatic (*E^elect^*), the AFM cross-correlation value between experimental AFM topography and calculated model (*CC^AFM^*), AFM potential (*V^AFM^*), AFM force factor (*θ^AFM^*), and estimated accuracy in terms of RMSD (pred), in Å.

| **Particle** | ***E^Total^*** | ***E^Local^*** | ***E^Go^*** | ***E^repul^*** | ***E^stacking^*** | ***E^pairing^*** | ***E^elect^*** | ***CC^AFM^*** | **V^AFM^** | **stage** | ***θ^AFM^*** | **pred** |
| --- | --- | --- | --- | --- | --- | --- | --- | --- | --- | --- | --- | --- |
| C1 | -9939 | 499 | -860.68 | 0.54 | -2237.71 | -7791.48 | 196.92 | 0.985 | 259.71 | -7.08 | 42 | 3.4 |
|  | -9962 | 488 | -856.42 | 0.88 | -2239.68 | -7801.2 | 194.93 | 0.985 | 254.59 | -5.29 | 42 | 3.5 |
|  | -9969 | 497 | -856.35 | 0.98 | -2244.42 | -7805.73 | 197.01 | 0.986 | 244.94 | -3.45 | 42 | 3.5 |
|  | -9941 | 513 | -863.15 | 0.95 | -2241.85 | -7798.76 | 195.64 | 0.985 | 257.97 | -5.37 | 42 | 3.5 |
|  | -9959 | 496.31 | -854.97 | 1.87 | -2244.74 | -7807.93 | 197.51 | 0.985 | 257.11 | -5.16 | 42 | 3.5 |
|  | -9959 | 494.35 | -854.99 | 1.95 | -2245.66 | -7803.93 | 196.96 | 0.985 | 255.34 | -4.45 | 42 | 3.5 |
|  | -9966 | 494.18 | -857.54 | 1.56 | -2244.65 | -7810.48 | 194.75 | 0.985 | 258.65 | -4.98 | 42 | 3.5 |
|  | -9931 | 507.24 | -850.49 | 1.56 | -2233.52 | -7809.87 | 198.39 | 0.985 | 259.62 | -5.19 | 42. | 3.5 |
|  | -9957 | 517.77 | -868.65 | 1.48 | -2243.51 | -7813.5 | 194.95 | 0.985 | 259.77 | -5.8 | 42 | 3.5 |
|  | -9939. | 499.56 | -860.68 | 0.54 | -2237.71 | -7791.48 | 196.92 | 0.985 | 259.71 | -7.08 | 42 | 3.4 |
| C2 | -10014.46 | 503.0 | -762.02 | 1.61 | -2881.36 | -7362.87 | 198.5 | 0.98 | 293.51 | -5.82 | 34 | 4.9 |
|  | -10043.62 | 482.52 | -761.25 | 1.34 | -2881.55 | -7367.79 | 197.48 | 0.98 | 289.05 | -4.39 | 34 | 4.9 |
|  | -10054.72 | 485.57 | -766.96 | 1.1 | -2886.0 | -7372.81 | 196.85 | 0.98 | 291.56 | -5.02 | 34 | 4.9 |
|  | -10039.31 | 486.83 | -757.34 | 1.4 | -2878.43 | -7370.01 | 197.64 | 0.98 | 281.92 | -2.3 | 34 | 4.9 |
|  | -10050.17 | 482.44 | -764.18 | 1.15 | -2884.59 | -7369.81 | 196.65 | 0.98 | 290.4 | -3.22 | 34 | 4.9 |
|  | -10059.15 | 489.47 | -777.96 | 1.48 | -2876.72 | -7373.54 | 194.71 | 0.98 | 291.15 | -8.72 | 34 | 5.1 |
|  | -10069.27 | 480.45 | -774.18 | 1.12 | -2879.81 | -7375.65 | 194.66 | 0.98 | 285.57 | -2.41 | 34 | 5.2 |
|  | -10026.39 | 485.07 | -749.23 | 1.06 | -2874.68 | -7373.56 | 196.36 | 0.98 | 292.23 | -4.62 | 34 | 5.2 |
|  | -10091.3 | 455.03 | -775.11 | 1.4 | -2876.29 | -7367.72 | 194.16 | 0.98 | 284.13 | -7.89 | 34 | 5.3 |
|  | -10038.16 | 503.86 | -768.83 | 1.77 | -2878.56 | -7370.47 | 195.32 | 0.98 | 282.2 | -4.43 | 34 | 5.3 |
| C3 | -10013.14 | 523.12 | -710.48 | 7.09 | -2873.12 | -7374.55 | 266.09 | 0.99 | 184.52 | -36.8 | 4 | 3.4 |
|  | -10036.87 | 503.51 | -704.13 | 6.97 | -2872.61 | -7363.82 | 262.62 | 0.99 | 167.36 | -37.77 | 38 | 3.5 |
|  | -10016.48 | 513.69 | -707.21 | 6.08 | -2877.23 | -7361.29 | 263.91 | 0.99 | 174.12 | -29.54 | 38 | 3.5 |
|  | -10061.97 | 521.15 | -708.26 | 5.49 | -2879.49 | -7364.37 | 265.32 | 0.98 | 132.82 | -35.63 | 25 | 3.5 |
|  | -10037.88 | 507.89 | -701.66 | 5.97 | -2871.84 | -7366.31 | 263.93 | 0.99 | 155.57 | -32.42 | 38 | 3.5 |
|  | -10024.67 | 526.09 | -706.59 | 6.07 | -2874.1 | -7362.31 | 265.38 | 0.99 | 152.62 | -32.82 | 38 | 3.5 |
|  | -10020.54 | 527.08 | -717.57 | 6.56 | -2881.91 | -7372.38 | 262.73 | 0.99 | 193.91 | -39.94 | 45 | 3.5 |
|  | -10026.17 | 520.23 | -706.83 | 5.83 | -2872.52 | -7360.56 | 264.0 | 0.99 | 154.86 | -32.18 | 38 | 3.5 |
|  | -10019.2 | 519.54 | -705.4 | 6.64 | -2879.88 | -7373.56 | 263.38 | 0.99 | 193.04 | -43.97 | 45 | 3.50 |
|  | -10034.14 | 528.52 | -698.23 | 7.4 | -2872.74 | -7375.47 | 266.32 | 0.99 | 144.92 | -35.85 | 38 | 3.5 |
| C4 | -9765.78 | 583.29 | -684.85 | 6.35 | -2881.76 | -7357.73 | 255.45 | 0.98 | 355.57 | -43.09 | 48 | 3.9 |
|  | -9775.34 | 585.93 | -678.89 | 5.27 | -2870.43 | -7367.19 | 259.18 | 0.98 | 333.13 | -43.31 | 48 | 3.9 |
|  | -9783.46 | 579.44 | -680.97 | 6.24 | -2879.34 | -7356.54 | 254.96 | 0.98 | 338.83 | -47.05 | 48 | 3.9 |
|  | -9791.82 | 582.83 | -672.34 | 6.31 | -2883.99 | -7362.21 | 258.13 | 0.98 | 328.56 | -50.09 | 48 | 3.9 |
|  | -9831.51 | 583.94 | -685.61 | 6.08 | -2880.96 | -7372.99 | 255.24 | 0.9812745 | 309.57 | -47.77 | 40 | 4.0 |
|  | -9786.37 | 576.01 | -685.73 | 5.98 | -2875.55 | -7362.65 | 253.07 | 0.98 | 345.02 | -43.51 | 48 | 4.0 |
|  | -9801.63 | 591.78 | -687.79 | 6.24 | -2884.07 | -7370.75 | 254.43 | 0.98 | 334.53 | -46.99 | 45 | 4.0 |
|  | -9769.09 | 592.48 | -680.61 | 5.73 | -2869.26 | -7360.89 | 256.53 | 0.98 | 332.73 | -46.79 | 48 | 4.0 |
|  | -9842.68 | 575.5 | -691.05 | 5.99 | -2876.93 | -7363.72 | 252.53 | 0.98 | 306.34 | -52.33 | 38 | 4.0 |
|  | -9765.78 | 583.29 | -684.85 | 6.35 | -2881.76 | -7357.73 | 255.45 | 0.98 | 355.57 | -43.09 | 48 | 4.0 |
| C5 | -9968.93 | 423.86 | -1035.55 | 0.75 | -2881.76 | -7357.73 | 255.45 | 0.98 | 355.57 | -43.09 | 48 | 3.9 |
|  | -9965.0 | 433.53 | -1027.5 | 0.71 | -2209.43 | -7582.66 | 207.67 | 0.9752032 | 225.47 | 0.0 | 22 | 3.9 |
|  | -9953.99 | 439.07 | -1033.2 | 1.3 | -2207.83 | -7578.91 | 208.54 | 0.9751414 | 205.48 | 0.0 | 20 | 4.0 |
|  | -9885.39 | 445.49 | -1032.62 | 1.01 | -2205.37 | -7591.39 | 207.99 | 0.9750753 | 226.63 | 0.0 | 22 | 4.0 |
|  | -9883.35 | 451.94 | -1027.12 | 0.5 | -2202.93 | -7590.48 | 207.11 | 0.9752811 | 286.06 | 0.0 | 28 | 4.1 |
|  | -9978.87 | 413.03 | -1035.1 | 0.72 | -2208.98 | -7571.73 | 207.58 | 0.975481 | 263.48 | 0.0 | 26 | 4.1 |
|  | -9893.2 | 432.42 | -1025.15 | 0.86 | -2207.04 | -7583.19 | 205.19 | 0.9750844 | 226.55 | 0.0 | 22 | 4.0719137 |
|  | -9911.8 | 431.64 | -1024.24 | 0.82 | -2214.94 | -7579.29 | 208.27 | 0.9754887 | 283.66 | 0.0 | 28 | 4.1 |
|  | -9921.5 | 436.29 | -1029.54 | 0.72 | -2211.92 | -7584.04 | 207.73 | 0.9751321 | 267.23 | 0.0 | 26 | 4.1 |
|  | -9968.93 | 423.86 | -1035.55 | 0.75 | -2211.78 | -7586.77 | 207.41 | 0.9756941 | 261.19 | 0.0 | 26 | 4.1 |

**Supplementary Table 8 |** Cutting limits applied to the dataset for the raw trajectory data. The procedure is performed by two successive filtering steps.

| Step | Component | Cutoff ($n\sigma_{E_{j}}$) |
| --- | --- | --- |
| 1 | $E^{Local}$ | ($-\infty$, 2) |
|  | $E^{repulsive}$ | ($-\infty$, 1) |
|  | $E^{stacking}$ | ($-\infty$, 1) |
|  | $E^{pairing}$ | ($-\infty$, 3) |
|  | $B^{stage}$ | ($-\infty$, 3) |
| 2 | $CC^{AFM}$ | (- 2, +$\infty$) |
|  | $E^{total}$ | ($-\infty$, 3) |

**Supplementary Table 9 | Cohort filtering selection.** Selection limits applied to the dataset for the particles populated in the selected cluster.

| Component | Cut ($n\sigma_{E_{j}}$) |
| --- | --- |
| $E^{Go}$ | ($-\infty$, 0) |
| $E^{Local}$ | ($-\infty$, 3) |
| $\mathrm{CC}^{AFM}$ | (0,+$\infty$) |
| $E^{Total}$ | ($-\infty$, -1) |

**Supplementary Table 10 | FARFAR2 inputs used to generate the structure models for RNase P RNA catalytic domain^86^ benchmarking.**

| RNA | Sequence | Secondary structure | Secondary structure | residue proximity |
| --- | --- | --- | --- | --- |
| RNase P  PDB ID: 3DHS | guuaaucaugcucggguaaucgcugcggccg  guuucggccguagaggaaaguccaugcucgc  acggugcugagaugccgcgagaaacccaaaugaugaugguaggggcaccuucccgaaggaaaugaacggagggaaggacaggcggcgcaugcagccuguagauagaugauuaccgccggaguacgaggcgcaaagccgcuugcaguacgaagguacagaacauggcuuauagagcaugauuaacguc | (((((((((((((.(((((((.(((((((((....)  )))))))).{.[.[[.[[[[[(((((((((.(((.  ..).)))))).....(((((....))))))))))...(  ((...........)))...(((((((...(........)...  )))))))((((((..........))))))........)))  ))))(((...((((..(((.....))).......))))..  .))).}....]]]]]]]]...)))))))))))))... | 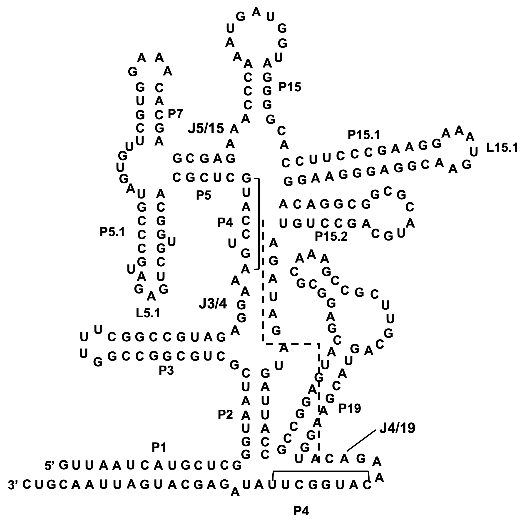 | 72-143  73-142  182-241  180-129 |

**
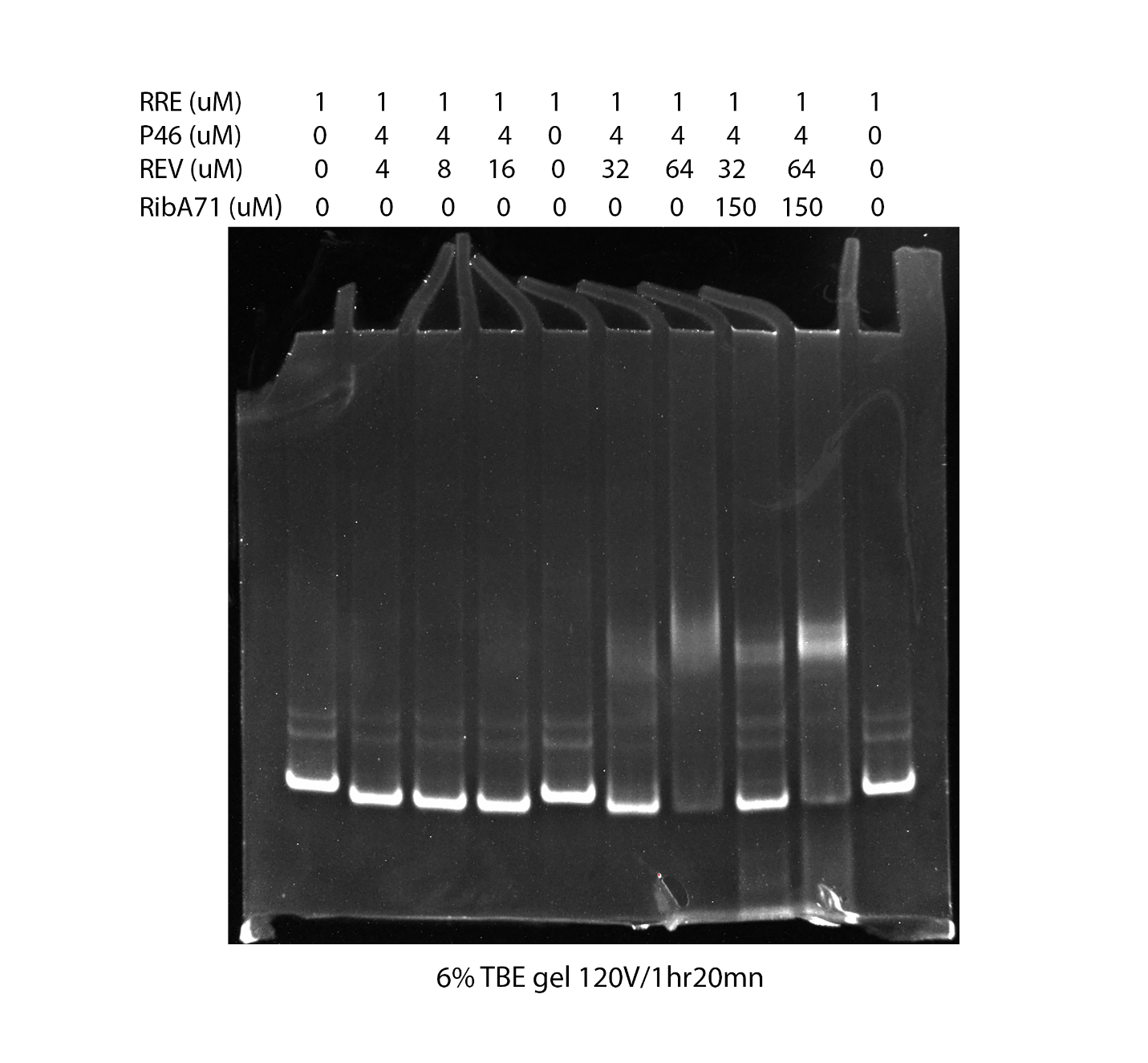
Supplementary Figure 1.** The raw gel image is annotated on both the top and bottom of the gel. The controls (RRE without the peptide P46, Rev, or RibA71 RNA) are Lanes 1, 5 and 10 counting from left to right.

**References**

74 Kenzaki, H. *et al.* CafeMol: A Coarse-Grained Biomolecular Simulator for Simulating Proteins at Work. *J Chem Theory Comput* **7**, 1979-1989 (2011). https://doi.org/10.1021/ct2001045

75 Niina, T., Fuchigami, S. & Takada, S. Flexible Fitting of Biomolecular Structures to Atomic Force Microscopy Images via Biased Molecular Simulations. *J Chem Theory Comput* **16**, 1349-1358 (2020). https://doi.org/10.1021/acs.jctc.9b00991

76 Cruz, J. A. & Westhof, E. The dynamic landscapes of RNA architecture. *Cell* **136**, 604-609 (2009). https://doi.org/10.1016/j.cell.2009.02.003

77 Bhandari, Y. R. *et al.* Topological Structure Determination of RNA Using Small-Angle X-Ray Scattering. *J Mol Biol* **429**, 3635-3649 (2017). https://doi.org/10.1016/j.jmb.2017.09.006

78 Martinez, H. M., Maizel, J. V., Jr. & Shapiro, B. A. RNA2D3D: a program for generating, viewing, and comparing 3-dimensional models of RNA. *J Biomol Struct Dyn* **25**, 669-683 (2008). https://doi.org/10.1080/07391102.2008.10531240

79 Schwieters, C. D., Kuszewski, J. J., Tjandra, N. & Clore, G. M. The Xplor-NIH NMR molecular structure determination package. *J Magn Reson* **160**, 65-73 (2003). https://doi.org/10.1016/s1090-7807(02)00014-9

80 Spiegel, M. R. & Stephens, L. J. *Schaum's Outline of Statistics*. 6th edition. edn, (McGraw-Hill Education, 2018).

81 Jolliffe, I. T. *Principal Component Analysis*. (2013).

82 Shlens, J. A Tutorial on Principal Component Analysis. (2014).

83 Mannor, S. *et al.* in *Encyclopedia of Machine Learning* 563-564 (Springer US, 2011).

84 Kingma, D. P. & Ba, J. L. in *ICLR* Vol. 9 15 (arXiv, 2017).

85 Reddi, S. J., Kale, S. & Kumar, S. in *ICLR* Vol. 1 23 (2019).

86 Kazantsev, A. V., Krivenko, A. A. & Pace, N. R. Mapping metal-binding sites in the catalytic domain of bacterial RNase P RNA. *RNA* **15**, 266-276 (2009). https://doi.org/10.1261/rna.1331809

87 Eismann, S. *et al.* Hierarchical, rotation-equivariant neural networks to select structural models of protein complexes. *Proteins* **89**, 493-501 (2021). https://doi.org/10.1002/prot.26033

88 Watkins, A. M., Rangan, R. & Das, R. FARFAR2: Improved De Novo Rosetta Prediction of Complex Global RNA Folds. *Structure* **28**, 963-976 e966 (2020). https://doi.org/10.1016/j.str.2020.05.011

89 Johnson, J. E., Jr., Reyes, F. E., Polaski, J. T. & Batey, R. T. B12 cofactors directly stabilize an mRNA regulatory switch. *Nature* **492**, 133-137 (2012). https://doi.org/10.1038/nature11607

90 Marcia, M. & Pyle, A. M. Visualizing Group II Intron Catalysis through the Stages of Splicing (vol 151, pg 497, 2012). *Cell* **151**, 1386-1386 (2012). https://doi.org/10.1016/j.cell.2012.11.037

91 Kazantsev, A. V. *et al.* Crystal structure of a bacterial ribonuclease P RNA. *Proc Natl Acad Sci U S A* **102**, 13392-13397 (2005). https://doi.org/10.1073/pnas.0506662102

92 Bhandari, Y. R., Jiang, W., Stahlberg, E. A., Stagno, J. R. & Wang, Y.-X. Modeling RNA topological structures using small angle X-ray scattering. *Methods* **103**, 18-24 (2016). https://doi.org/https://doi.org/10.1016/j.ymeth.2016.04.015
